# Gender Disparities in Cardiovascular Disease and Their Management: A Review

**DOI:** 10.7759/cureus.59663

**Published:** 2024-05-05

**Authors:** Dhruva Betai, Aamina S Ahmed, Prerna Saxena, Hurria Rashid, Happy Patel, Atika Shahzadi, Adetola G Mowo-wale, Zahra Nazir

**Affiliations:** 1 General Practice, Pandit Deendayal Upadhyay Medical College, Rajkot, IND; 2 Internal Medicine, St. George's University School of Medicine, New York, USA; 3 Medicine and Surgery, K. S. Hegde Medical Academy, Mangalore, IND; 4 Basic Sciences, Fatima Jinnah Medical University, Lahore, PAK; 5 Internal Medicine, Angeles University Foundation, Angeles City, PHL; 6 Medicine, Aziz Bhatti Shaheed Teaching Hospital, Gujrat, PAK; 7 Internal Medicine, Obafemi Awolowo College of Health Sciences, Sagamu, NGA; 8 Internal Medicine, Combined Military Hospital Quetta, Quetta, PAK

**Keywords:** add-on pharmacotherapy, health aging, ecg abnormalities, gender role, cardiology corner

## Abstract

Worldwide, cardiovascular diseases (CVDs) are still the primary cause of death, and there are notable differences between sexes when it comes to symptoms/course and treatment. Due to evolving healthcare technologies, significant progress has been made in understanding CVDs. Hence, it is evident that gender disparities exist in the clinical presentation, prevalence, management, outcomes, and risk factors, including biological, behavioral, and sociocultural factors. This narrative review is designed to provide a generalized idea of gender disparities in CVDs. It aims to provide insights to prove the role of hormonal influences, genetic predispositions, and the difference in physiological outcomes owing to different genders. This review explores subtle distinctions in CVD across genders, including changes in structure, biology, and hormones that affect how illness presents and progresses. Lifestyle variables also influence sociocultural factors and gender disparities in risk profiles. Traditional risk factors, diabetes mellitus (DM), cholesterol levels, and smoking may have different weights and relevance in men and women. Moreover, age and other conventional risk variables have distinct effects on gender. Treatment efficacy may be impacted by the expression of gender-specific factors, emphasizing the necessity for customized strategies. Development of CVDs can be delayed or prevented, and its consequences can be lessened with the early identification and effective management of gender-specific factors. More investigation is necessary to clarify complex interactions between structural, biochemical, and hormonal aspects across genders in order to maximize treatment results and reduce the burden of CVDs.

## Introduction and background

Cardiovascular diseases (CVDs) are a prevalent health concern and a leading cause of mortality worldwide. CVDs are disorders of the heart and blood vessels [1]. Coronary heart disease affects the heart arteries, while cerebrovascular disease impacts brain arteries. Peripheral arterial disease affects blood vessels in the arms and legs. Rheumatic heart disease damages the heart muscle and valves due to untreated streptococcal infection. Congenital heart disease involves structural heart defects from birth. Deep vein thrombosis forms leg blood clots, and pulmonary embolism occurs when these clots travel to the heart or lungs [2]. CVDs account for 32% of deaths worldwide; of these deaths, 85% were due to heart attack and stroke [3]. The prevalence of CVDs varies, and cardiovascular deaths as a percentage of total deaths and total population in seven economic regions of the world as defined by the World Bank comprises of the following: North America/US/UK/Australia (32.5%), Latin America and the Caribbean (27.1%), Middle East and North America (44.3%), Sub-Saharan Africa (13.8%), South Asia (27.6%), East Asia and Pacific (38.8%), Europe and Central Asia (44.7%) [2]. CVDs impact both high-income and low-to-middle-income countries and cause a considerable economic burden on healthcare systems.

Smoking, obesity, diabetes, hypertension, and hyperlipidemia predispose patients to CVDs [1]. Tobacco use damages blood vessels, building fatty deposits known as atherosclerotic plaques in the arteries. This chemical change promotes inflammation and causes blood clot formation, increasing cardiovascular complications. Obesity is linked with insulin resistance, dyslipidemia, and hypertension, leading to conditions, such as atrial fibrillation and heart failure. Diabetes leading to high blood sugar levels damages blood vessels and nerves. This condition also affects the balance of cholesterol, increasing low-density lipoproteins (LDL) and decreasing high-density lipoproteins (HDL), which increases the risk of myocardial infarctions and strokes. Hypertension places a strain on the heart and blood vessels. Over time, this can lead to the hardening of the arterial walls, damaging critical organs, such as the heart, kidneys, brain, and eyes. Hyperlipidemia due to elevated cholesterol levels and triglycerides can also lead to inflammation and buildup of plaques damaging endothelial arteries. Lifestyle modifications and public health interventions can prevent these behavioral risk factors.

Each gender manifests CVDs differently, which can significantly affect the disease process. These can be categorized into structural, biological/hormonal differences, disease progression, and response to therapy and outcomes. Structural differences are accounted for by the variation in heart sizes between males and females. Females have smaller heart sizes and blood vessels, making them more prone to developing plaques. Hormonal differences in estrogen, progesterone, and testosterone also impact CVDs in each gender. Estrogen is protective for pre-menopausal women, and a drop in estrogen levels in menopausal women increases cardiovascular incidents [3]. The progression of CVDs differs significantly between each gender.

Males present with heavily calcified plaques, which causes high-grade obstruction. Females have less calcified plaques but are more prone to rupture, resulting in widespread diseases [4]. Therapeutic modalities, such as ACE inhibitors, have also been more efficacious in females than males [5]. Research studies have shown that the combination of beta-blockers/diuretics is particularly beneficial in women [6]. Females, however, are less likely to receive invasive treatments for CVDs, such as catheterization and revascularization, because they have higher complication rates. Smaller vessels, increased bleeding risk, advanced disease, and comorbidities such as diabetes. Hypertension and cerebrovascular disease are associated with increased complications in women [3]. The manifestation and development of gender-specific risk factors can influence the effectiveness of treatments for CVDs. Therefore, additional research is warranted to elucidate the structural, biological, and hormonal distinctions between genders to improve therapeutic outcomes for CVDs.

This study brings into focus several gender-specific risk factors that play a role in the presentation of CVDs and their progression. A thorough understanding of these gender disparities can augment the treatment of CVDs in clinical practice. Early detection and appropriate management of these gender-specific risk factors can help prevent or delay the onset of CVDs and its complications.

## Review

Studies are being done to understand various aspects of CVDs, such as their incidence, prevalence, pathophysiology, risk factors, symptoms, outcomes, and prognosis. However, early studies placed less emphasis on sex- and gender-specific differences within these aspects. Initially, research predominantly focused on male populations, partly due to the lower enrollment of women in clinical trials. This discrepancy led to a lack of evidence regarding sex- and gender-specific outcomes, culminating in assumptions about the treatment and prognosis of CVDs in women. Consequently, while men experienced improvements in CVD management due to adequate research, women's health declined due to a lack of targeted evidence [7].

Recent studies have successfully established the importance of sex- and gender-specific differences in CVDs. In the past decade, increased initiatives have emerged to bridge this knowledge gap. The Federal and American Heart Association (AHA) launched several campaigns with increased women's participation. Between 1997 and 2007, the perception of heart disease as the leading cause of death among women doubled, and the death rate from CVDs nearly halved [8].

There has been a shift toward gender-sensitive approaches in research, providing equal opportunities to explore gender disparities in CVDs. This shift has led to improved treatment modalities for both men and women. Currently, there is an abundance of published articles on gender disparities in CVDs; however, the knowledge remains dispersed and unorganized.

Prevalence and presentation of CVDs in different genders

The frequency of CVDs varies significantly between each gender, and differences can be noted in the prevalence, symptoms, and outcomes of specific cardiovascular conditions, such as chronic heart disease (CHD), stroke, hypertension, heart failure, and peripheral arterial diseases between men and women.

CHD is more prevalent in men than women, with a prevalence of 8.3% in men and 6.1% in women [8]. However, after menopause, this difference diminishes in women. Women with CHD also present with altered symptoms compared to men, consisting of atypical chest pain, nausea, fatigue, and shortness of breath, leading to undertreatment. The prevalence of stroke is greater in women than men, with a prevalence of 2.7% in men versus 3.3% in women [8]. Women also experience worse symptoms and prognosis compared to men.

Hypertension affects both men and women similarly, and the prevalence is nearly equal in both genders. Research has shown these numbers to vary based on age and ethnicity. The prevalence of high blood pressure was found to be greater in women aged >65, with the highest rate of 44% in black women [8]. Women also presented differently compared to men, with increased rates of isolated hypertension, which made them more prone to CVDs.

Heart failure has also been found to be comparable between both genders, with a prevalence rate of 3.0 in men compared to 2.0 in women [8]. Women are more predisposed to develop heart failure with preserved ejection fraction (HFpEF), whereas heart failure with reduced ejection fraction (HFrEF) can occur more in men [9].

Peripheral arterial disease occurs more in men compared to women. Women with peripheral arterial disease are more likely to have an atypical presentation with less severe symptoms compared to men [9,10]. 

Gender-specific risk factors involved in CVDs

Prevention of CVDs can be done by identifying the risk factors involved and taking measures to decrease them. Some common risk factors include obesity, hypertension, diabetes, raised LDL levels, and smoking [3]. However, the contribution of risk factors is gender specific and can be studied, as shown in Table 1:

**Table 1:** Risk factors in females increasing CVD risk Source reference: [10,11] CVD: cardiovascular disease, PCOS: polycystic ovarian syndrome, T2DM: type 2 diabetes mellitus

Female risk factors	Description
Pregnancy	Pregnancy contributes to various risk factors involved in CVDs, like gestational hypertension (relative risk of 2.1 as compared to women with a normotensive pregnancy), gestational diabetes (relative risk of 7.4 as compared to women with a normal pregnancy). In addition, pregnancy is a prothrombotic state that predisposes to an increased risk of venous thromboembolism contributing to CVD and CVA. Adverse pregnancy outcomes and fetal and placental complications like stillbirth also increase the risk of CVDs [10].
Menopause	The female sex hormone estrogen is proven to be effective in the prevention of CVDs due to its anti-lipid property. During menopause, the sex hormones decrease and hence the protective effect also decreases, thereby increasing the risk of CVDs [10].
PCOS	PCOS is a state with raised androgen levels leading to ovarian dysfunction, thereby causing insulin resistance. Insulin resistance causes problems like T2DM, chronic hypertension, and dyslipidemia [10].
Genetics	Regulation of the X chromosome is of special interest in diseases that have a different prevalence between sexes. Females have 2 X chromosomes while males have only one. The X chromosome regulates gene expression between different sexes and individuals. It contains information involved in inflammation and contributes largely to autoimmune diseases that are highly female-specific. Autoimmune diseases cause a significant threat and an increased risk of CVDs [10].
Cardiovascular anatomy	Females have a microvascular arterial structure compared to their male counterparts. Due to this microvascular anatomy, the plaque stabilizes in these arteries, thereby increasing the risk of a heart attack. Meanwhile, in males, the plaque does not stabilize, so the chances of plaque rupture or an embolism are higher in men [10].
Psychological	Factors like stress, anxiety, and depression are predominantly found more in females than males, thereby increasing the risk of CVDs in females [11].
Social	Lack of awareness regarding the risk factors, low levels of education, late diagnosis of CAD, greater clustering of risk factors, less opportunity for optimal treatment, low physical activity, low magnitude of lifestyle changes, and no attainment of therapeutic goals due to gender-specific roles attributed by the society to women [11].

Men: risk factors involved

**Figure 1:**
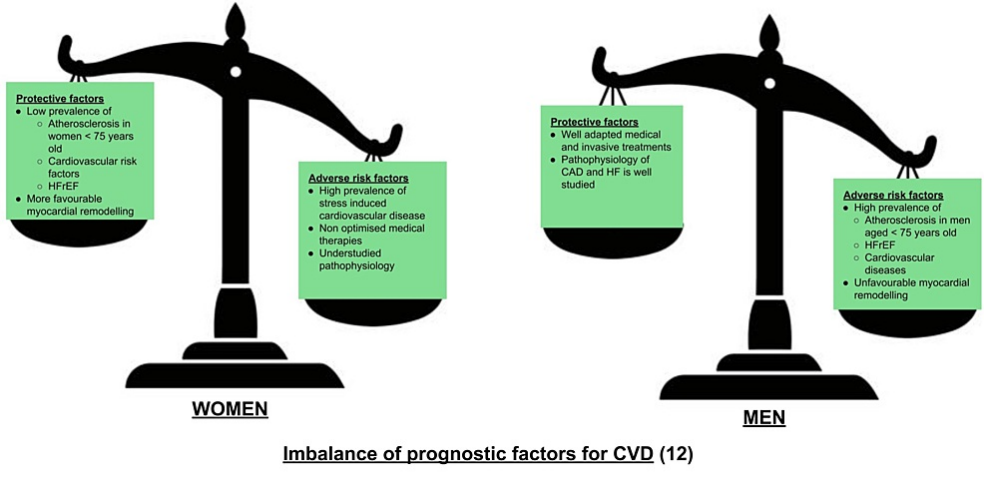
Imbalance of prognostic factors of CVDs in men and women Source reference: [12,13] HFrEF: heart failure with reduced ejection fraction, HF: heart failure, CAD: coronary artery disease

Metabolic syndromes, regular smoking, prostate cancer, and central obesity contribute to an increased risk of CVDs, primarily in males [11]. Figure 1 depicts the imbalance in prognostic factors among men and women.

Biological factors contributing to gender disparities 

Males and females have significant anatomical and functional differences in their cardiovascular system. It is observed that females have smaller heart sizes, smaller blood vessels, and increased ventricular contractility compared to their male counterparts [12]. Generally, the heart size in women is 0.25 times smaller than in men. A smaller heart contributes to a smaller left ventricle in females, resulting in a decreased end-diastolic volume, end-systolic volume, and a decreased stroke volume that results in an increased heart rate in women compared to men. Due to the small vessel size, there is an increased risk of hospital mortality in patients undergoing coronary artery bypass grafting. Hence, gender plays a vital role in predicting the size of coronary vessels. Due to the discrepancies in the heart's anatomy in men and women, we can expect an appropriate cardiovascular response but with different outcomes [13]. For example, in conditions that cause a significant amount of stress on the cardiovascular system, like exercise or any psychological stress, males combat it by increasing the total peripheral resistance of the vessels. Females respond by increasing their heart rate, so cases of raised blood pressure in acute stress are seen more commonly in males while fainting due to orthostatic hypertension is seen more commonly in females. Different outcomes when exposed to the same amount of stress are due to different mechanisms of the baroreflex system in men and women. In women, the sympathetic nervous system activity decreases, causing a reduction in total peripheral resistance. By contrast, in men, the sympathetic nervous system activity increases, which is proven by increased plasma norepinephrine levels, attributing to increased total peripheral resistance. Hence, women are more vulnerable to orthostatic hypotension and fainting when exposed to the same amount of stress as men [14].

The female sex hormone estrogen is proven to be protective against CVDs due to the anti-lipid effect of estrogen as it decreases LDL, lipoprotein A, homocysteine, and fibrinogen levels. At the same time, it increases HDL and fibrinolysis, which is attributed to its antioxidant properties, thereby improving endothelial function. Hence, premenopausal women have a lower risk of CVDs, and post-menopausal women are at increased risk of cardiovascular events due to diminished estrogen levels [15]. Men, unlike women, do not experience an abrupt decrease in sex hormones, but an age-associated gradual decrease does happen. Testosterone and dehydroepiandrosterone (DHEA) play a significant role in increasing HDL levels and decreasing triglyceride and LDL levels. Due to increasing age, the effects gradually decrease, thereby posing a higher risk of CVDs [16].

The role of genetic risk in CVDs is more in women with a strong family history as compared to men. Coronary artery disease (CAD) is heritable with a vital genetic component. Premature death due to cardiovascular complications, such as heart attack, is quite common in patients who have a strong paternal history as compared to a maternal history [17-20].

Differences in lifestyle factors

Irrespective of the commonly shared features among both genders, the significance and impact of each risk factor varies. Increasing age, elevated blood pressure, high cholesterol, and LDL-cholesterol greatly influence men. However, smoking, diabetes, triglyceride, and HDL-cholesterol levels mainly affect women [21].

Traditional risk factors

Aging is a significant factor, but it varies by gender. In men, the risk profile of cardiac events linearly increases over time, and the atherosclerotic process is constantly evolving, while women of fertile age are protected from atherosclerosis due to the beneficial effects of estrogen on the cardiovascular system. However, the incidence of stroke is higher among menopausal women [22].

Many large-scale analyses have shown that the link between body mass index (BMI) and CHD is broadly identical between men and women. By contrast, the risk for stroke with an increase in BMI may be higher in men than in women. An increase in BMI of up to 5 kg/m^2^ led to an increased risk for CHD of 1.35 in women and 1.42 in men and an increased risk for fatal stroke of 1.30 in women and 1.50 in men [23].

Smoking is one of the typical causes of CVDs, causing nearly six million deaths per year. It has been found that at younger ages (<50 years), smoking is more deleterious in women than in men. Smoking is associated with a greater risk of a first acute myocardial infarction (AMI) in women than in men [24]. It may be due to smoking causing downregulation of the estrogen-dependent vasodilatation of the endothelial wall.

CVDs have a strong association with hypertension and are an essential cause of left ventricular hypertrophy (concentric), diastolic heart failure, and stroke. Systolic BP is higher in young men than young females [25].

Elevated LDC cholesterol (LDL-C) is more likely to increase cardiovascular risk in men than in women. By contrast, HDL cholesterol (HDL-C) levels mainly act in women [26]. According to a meta-analysis, in both men and women, each ​mmol/L total cholesterol reduction can reduce the risk of mortality from CHD, with about a half lower in early middle age (40-49 years), about a third lower half in later middle age (50-69 years old), and about a sixth lower in old age (70-89 years old) [27].

Diabetes increases CHD risk by threefold to sevenfold in women and by twofold to threefold in men [28]. Heart failure is also strongly related to diabetes since its prevalence can cause heart failure by 40% [29]. The gender difference in HF risk, first shown in the Framingham Heart Study, was that men were two times more likely to have heart failure (P ​< ​0.05), compared with five times in women with the respective non-diabetic population (P ​< ​0.01) [30].

Besides the traditional risk factors, there are unique gender-specific risk factors in women, such as premature menopause, chronic inflammatory diseases such as systemic lupus erythematosus (SLE), and pregnancy complications like preeclampsia increase the risk for CVDs. During postmenopausal, the incidence of CAD starts to increase exponentially in women.

Women who suffer from gestational hypertension are more prone to develop hypertension and premature CVD later in life. Women with a history of preeclampsia are twice as likely to develop CHD compared to women who are normotensive during pregnancy. Women with gestational diabetes are seven to 12 times more likely to develop type II DM when compared with women who have normoglycemic pregnancies. Women with autoimmune diseases like SLE and RA are also at a greater for CHD.

Research shows that women diagnosed with CVD receive less intensive screening and treatment compared to men and are less scheduled to undergo cardiac procedures.

Gender disparities in diagnostic procedures and tools

There is evidence to suggest the presence of gender disparities in diagnostic procedures and tools. Diagnostic accuracy is lower in women than in men [31].

Electrocardiography (ECG)

Exercise electrocardiography (ECG) is one of the initial options for identifying CVDs, as per the American College of Cardiology (ACC)/AHA [32]. While performing exercise stress tests for diagnosing CVDs, there have been several gender-specific differences. Although the accuracy of this test in women remains lower than in men, it can be improved using other gender-specific criteria [32,33]. Lower accuracy in women is attributed to lower CAD prevalence, inability to achieve maximum levels of exercise (not more than five metabolic equivalents of treadmill exercise), more frequent ST-T wave changes, lower ECG voltage, hormonal factors in women, and false-positive ST-segment responses [33-35].

In a published study, when different criteria were used, such as standard ST segment depression and simple magnitude of ST depression, different outcomes were found. Using the standard criteria, the specificity was similar in men and women (96%), with different sensitivities in 67% of men and 51% of women, which remained the same when gender-specific criteria were used. When using the simple magnitude of ST depression criteria, there was a disparity in the sensitivity results seen in both genders. Considering gender-specific criteria, specificity was 96% in both genders, whereas sensitivity was reduced in men (60% -> 55%) and increased in women (30% -> 35%) [32].

Although specificity remains similar in both genders, sensitivity differs. Integrating multiple criteria, such as ST/heart rate index, Duke treadmill score, and functional capacity, can potentially improve these tests' diagnostic performance and accuracy [32,33].

Stress-induced perfusion abnormality assessment

During the evolution of ischemic attack, the first event to be noticed was reduced myocardial perfusion, even before other events, such as ECG abnormalities. Thus, single-photon emission computed tomography (SPECT) is done to look for myocardial perfusion abnormalities as it may have high sensitivity in both men and women [35,36]. However, the accuracy of the SPECT test in women is lower as compared to men because of the small heart in women that provides limited myocardial area, and thus, the tiny areas with reduced perfusion may be missed due to limitations in spatial resolutions of the cameras. Another factor in women is the variable breast tissue attenuation [35,37]. However, higher-energy Tc-99m radioisotopes are used in women, which significantly improves diagnostic specificity in women [35]. 

Impact of gender bias on clinical decision-making

As women are underrepresented in clinical trials, it results in selection bias. Other forms of gender bias are seen in diagnosis and treatment as well. The diagnosis of CVDs in women remains a challenge, in contrast to the diagnosis of CVDs in men. Gender-based differences in epidemiology and risk factors keep women at less risk of developing CVDs than men up until the women’s seventh decade of life [33].

Significant referral bias was observed in the management of CVDs. Women are less likely to be referred for cardiac rehabilitation and revascularization than men for ST-segment elevation myocardial infarction (STEMI), non-STEMI (NSTEMI), and stable angina, which is seen even more in younger women [38].

Women are less likely to receive primary and secondary preventive therapy, and even if they do, therapies are primarily evidence-based, and appropriate preventive therapy guidelines are still not followed [38].

Differences in response to various treatments (e.g., pharmacological and surgical)

Since the prevalence of CVDs in women is often underestimated, the strategies for treatment approach are usually less aggressive [39]. Widespread gender differences are seen in CVDs, so it is worth noting the gender impact on the treatment of CVDs. Newer guidelines more specifically for women have been formulated by the AHA, which certainly improved the overall management and treatment of CVDs in women [40].

Still, there are higher case fatality rates seen in women as compared to men due to multiple factors, such as less awareness, delayed hospitalization, higher clustering cardiovascular risk factors, inadequate diagnosis and hospitalization, and less aggressive approaches. Women undergo referral bias, and they receive less intensive pharmacotherapy for both treatment and prevention of CVDs, which eventually leads to suboptimal outcomes in women [38,41].

Higher morbidity and case fatality in women can also be attributed to increased post-intervention complications seen in women and a higher incidence of bleeding leading to cardiogenic shock (5.8% vs. 4.0%) and heart failure (5.8% vs. 3.4%) compared with men [38]. Adjusted in-hospital mortality rates in younger women (<60 years) are nearly two times higher than in men [41]. Furthermore, these complications may be justified due to variable biological responses to pharmacological and reperfusion treatment and less hospital and post-discharge care.

Pharmacotherapy

Statins, aspirin, and beta-blockers are equally efficient in secondary prevention in men and women [38]. The risk of heart failure after MI in valsartan is higher in women, according to a trial named VALIANT [41].

Surgical interventions

Both men and women have the same benefits from percutaneous coronary intervention (PCI). However, women experience more peri-procedural MI and bleeding events post-PCI procedure. Residual symptoms of angina post-intervention are more often present in women than men, primarily due to more functional coronary abnormalities and diffuse patterns of atherosclerosis [41].

The use of trans-radial access for coronary interventions usually reduces peri-procedural bleeding complications, However, this method does not change the scenario in females and is more challenging due to the narrow lumen or radial artery and higher tendency to spasm [41].

According to a three-year follow-up of the FAME 3 trial, fractional flow reserve-guided PCI with current-generation drug-eluting stents does not show any difference in the incidence of death, MI, or stroke compared with coronary artery bypass grafting (CABG) [42]. However, the FFR-PCI has improved outcomes somewhat, and the FFR values are higher in women [41].

Procedures like cardiac resynchronization therapy (CRT) used for the treatment of heart failure patients with conduction delay also have some gender-specific outcomes. Few studies show that women experience more significant benefits independent of factors like QRS complex duration and baseline characteristics [41].

The International Society of Heart and Lung Transplantation (ISHLT) analysis shows that women survive less after device implantation such as a ventricular assist device. The analysis showed that the cause for this is the late referral of women to clinics. In other cases of transplantation, such as heart transplants, women play a major role as donors instead of being recipients. Women undergo heart transplantation less frequently than men, primarily due to referral bias. In cases of transcatheter aortic valve implantation (TAVI), although the peri-procedural mortality and complications are more in women, the survival rates are higher in women [41].

Hypertensive women have several critical findings as part of treatment outcomes. The chances of residual hypertrophy and reduced response to medical therapy are higher in women than in men. Hypertensive women develop vascular and myocardial stiffness as they age. All these factors increase the risk of developing congestive heart failure and stroke way more (three times) in women than men [41].

Outcomes and prognosis

A study found a significant difference between men and women in following post-discharge plans after being diagnosed and managed with the diagnosis of acute coronary syndrome. It showed that women were less compliant with attending cardiac rehabilitation after six months of discharge when compared to men. In addition, the usage of secondary prevention medications was the same among both sexes. Women were found to have more major adverse cardiovascular events, such as MI, stroke, and heart failure, when compared to men. However, there was no significant difference among them in the aspect of rates of all-cause death after six months and 12 months post-discharge from the hospital [43]. The role of estrogens in women has been studied to have some protective effect on the cardiovascular system, specifically vascular endothelium. Estrogen is an essential factor in the development of coronary disease in women, who are much older than men on average at the time of onset of coronary diseases. It was suggested by a study that as the frequency of depressive symptoms increased in women, the advantage of being female, i.e., the protective effects of estrogen, were diminished. We can imply that emotional well-being can be a potential aspect to target for preventing and improving any outcomes in patients with congestive heart disease, especially in cases of women [44,45].

In terms of neurologic outcomes, women were less likely to have an excellent neurologic prognosis at discharge from the hospital and after six months of cardiac arrest. Neurologic imaging and other neurophysiologic testing were similar among both sexes. In addition, women were more likely to have higher odds of undergoing withdrawal from life-sustaining therapy when compared to men [46]. As mentioned earlier, with women having poor neurologic prognosis, in a study, it was found that post-cardiac arrest, women had lower survival rates from admission to discharge when compared to men. The prognosis of PCI after out-of-hospital cardiac arrest (OHCA) was not significantly different between males and females. They also suggested that women are less likely to receive post-admission interventions, including PCI and coronary angiography, leading to poor survival in females post-OHCA. However, to improve the outcome, new insights on management are directed toward narrowing the difference between men and women in terms of prognosis after an OHCA [47].

Other than the findings mentioned earlier, it was found in a study that healthcare disparities, as well as mortality, can be improved in women with STEMI by developing required treatment protocols and systems in healthcare facilities. Hence, a PCI STEMI system, along with the standardized STEMI protocol and reduced treatment disparities among men and women, suggested that being a woman might not be a predictor of mortality [45].

## Conclusions

Gender disparities in CVDs are pretty obvious, emphasizing the essential differences in disease prevalence, risk factors, clinical presentation, and outcomes between both genders. Despite the evolution of healthcare, these differences continue to linger on, requiring a focused approach to identifying the risk factors, their prevention, diagnosis, and treatment. Dealing with this problem requires a multifaceted approach; this includes spreading awareness by educating healthcare providers and the community regarding the disparity in CVDs in both genders and making specific changes in the healthcare system, like improving diagnostic and treatment guidelines to warrant unbiased cardiovascular care for both genders.
